# A Critical Role of Neutrophil-Driven Amplification of Chronic Microinflammation in the Biocompatibility of Hemodialysis

**DOI:** 10.3390/ijms26136472

**Published:** 2025-07-04

**Authors:** Masaaki Nakayama, Hiroyuki Miyakawa, Kazuya Ohama, Hirokazu Kimura

**Affiliations:** 1 Division of Research Control, St Luke’s International University, Tokyo 104-0044, Japan; 2 Head Office for Open Innovation Business Development Strategy, Tohoku University, Sendai 980-8575, Japan; 3 Graduate School of Health Science, Gunma Paz University, Takasaki 370-0006, Japan; miyakawa@paz.ac.jp (H.M.); oohama@paz.ac.jp (K.O.); h-kimura@paz.ac.jp (H.K.)

**Keywords:** hemodialysis, biocompatibility, complement, NETosis, interleukin-8, microinflammation, interleukin-6

## Abstract

This review highlights recent insights into the pathophysiology and therapeutic strategies for improving biocompatibility in hemodialysis. Hemodialysis activates the innate immune system, particularly the complement cascade and neutrophils, leading to acute microinflammation. Interleukin-8 (IL-8), which increases during dialysis, promotes neutrophil chemotaxis and neutrophil extracellular trap (NET) formation, triggering myeloperoxidase (MPO) release and oxidative stress. Neutrophil accumulation in atherosclerotic plaques exacerbates vascular inflammation through IL-6 upregulation. Elevated levels of IL-8, MPO, and NET-related biomarkers are associated with increased all-cause and cardiovascular mortality in dialysis patients. Strategies to mitigate these effects include the use of advanced membrane materials (e.g., AN69, vitamin E-coated, polymethyl methacrylate), novel dialysis modalities (e.g., high-volume online hemodiafiltration, cool dialysate, hydrogen-enriched dialysate), and citrate-based anticoagulation. These approaches aim to suppress complement activation, reduce oxidative stress, and limit neutrophil-induced damage. Enhancing biocompatibility is crucial for reducing cardiovascular complications and improving outcomes in dialysis patients. Suppressing the innate immune response during dialysis may become a future cornerstone in extracorporeal blood purification therapy.

## 1. Introduction

Biocompatibility in hemodialysis represents a long-standing yet continuously evolving clinical challenge [1,2]. During hemodialysis, blood comes into contact with various foreign materials, including tubing, hemodialysis membranes (dialyzers), and dialysis fluid, which can stimulate leukocytes and platelets, subsequently activating or inducing various plasma proteins. The extent of such biological responses defines the concept of biocompatibility (Table 1). Immediate reactions may include complement system activation by the hemodialysis membrane, potentially resulting in hypotension or anaphylactic reactions, allergic responses related to ethylene oxide sterilization of dialyzers, and circuit occlusion due to platelet activation and thrombus formation. Persistent low-grade inflammation may further lead to long-term complications such as the progression of atherosclerosis, dialysis-related amyloidosis, and increased susceptibility to infections. In response to these challenges, advances in membrane materials and the widespread adoption of online hemodiafiltration (OL-HDF) have substantially improved the biocompatibility of dialysis treatments. However, it remains an open question whether this issue has been fully resolved from a clinical perspective.

**Figure 1:**
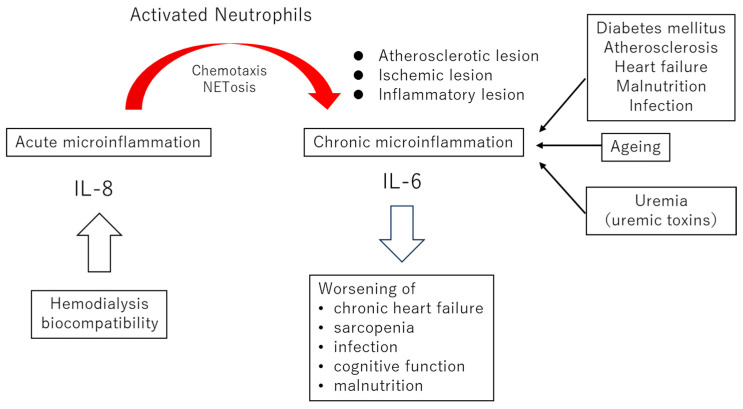
The impact of biocompatibility on chronic microinflammation. When neutrophils are stimulated during hemodialysis, they undergo chemotaxis toward sites of systemic atherosclerosis, ischemia, and inflammation. In this process, neutrophils produce interleukin-8 (IL-8), forming a positive feedback loop that further enhances neutrophil chemotaxis. At the sites of infiltration, activated neutrophils stimulate interleukin-6 (IL-6) production from monocytes and macrophages. In other words, the biocompatibility of dialysis triggers acute microinflammation, which in turn amplifies and worsens local chronic microinflammation at distant lesion sites. Clinically, poor biocompatibility may increase the risk of exacerbating heart failure, sarcopenia, susceptibility to infections, cognitive impairment, and malnutrition.

In this narrative review, we aim to provide a comprehensive overview of the latest pathophysiological insights and therapeutic strategies related to biocompatibility in hemodialysis. Based on current findings, we propose that contemporary issues regarding hemodialysis biocompatibility lie in its potential to amplify subclinical inflammation associated with underlying disorders in individual dialysis patients (Figure 1). Although classical immediate-type acute immune reactions have been largely controlled, activation of the complement system and subsequent neutrophil activation during dialysis are not fully suppressed. This response, which induces neutrophil extracellular trap (NET) formation (NETosis), is thought to play a pivotal role in amplifying inflammation in patients with subclinical inflammation, who represent the majority of the real-world dialysis population.

## 2. Pathophysiologic Aspects of Biocompatibility of Hemodialysis: Recent Advances and Clinical Significance

### 2.1. Commonalities of Chronic Microinflammation in the General Population and Dialysis Patients

Epidemiological studies in the general population have revealed associations between chronic microinflammation, oxidative stresses, and various metabolic disorders such as diabetes mellitus, atherosclerosis, chronic heart failure, malnutrition, and frailty [3,4,5,6]. In particular, elevated serum interleukin-6 (IL-6) levels have been identified as a prognostic marker in these populations [4,5,6]. Furthermore, an age-related persistent increase in proinflammatory cytokines, including tumor necrosis factor-alpha (TNF-α), IL-6, and IL-1β, that is referred to as inflammaging has been reported to increase the risk of these conditions [7,8]. The contemporary dialysis population increasingly includes elderly patients and individuals with comorbidities such as diabetes and obesity [9,10], who are high risk of atherosclerosis. Therefore, it is presumed that chronic inflammation in dialysis patients results not only from uremia-specific factors, such as uremic toxins [11], but also from the same pathological processes observed in the general population. Considering that IL-6 is a risk factor for mortality in dialysis patients [12], as it is in the general population [4,5,6], it may be critically important to mitigate inflammation in this population to prevent the progression of atherosclerotic lesions and improve overall prognosis.

### 2.2. The Roles of Neutrophil NETosis in Microinflammation Associated with Atherosclerosis

Neutrophil extracellular trap formation (NETosis) is an immune response in which neutrophils release web-like extracellular structures composed of their own DNA, histones, and antimicrobial proteins (such as myeloperoxidase and elastase) in response to various pathogens or foreign substances. These structures, known as neutrophil extracellular traps (NETs) may serve to entrap and neutralize invading microbes [13]. Since its initial report in 2004, NETosis has been increasingly recognized as a critical host defense mechanism [13]. Accumulating evidence suggests that this process is also involved with the pathogenesis of various conditions, such as autoimmune diseases, atherosclerosis, diabetes, thrombus formation, and cancer metastasis [14]. Recent studies suggest that NETosis plays a critical role for all phases of atherosclerotic development, from early lesion formation to progression and exacerbation [15]. Components of NETs, such as histones and fragmented DNA, act as damage-associated molecular patterns (DAMPs) that stimulate the expression of proinflammatory cytokines like interleukin-6 (IL-6) through the activation of pattern recognition receptors, such as Toll-like receptor 9, on immune cells including macrophages and dendritic cells [16,17]. Therefore, NETosis is hypothesized to exacerbate existing inflammatory conditions. In patients undergoing dialysis, enhanced NETosis has also been documented [18,19,20,21,22,23]. Increases in NETosis markers after dialysis [18,19], as well as correlations between these markers and various inflammatory biomarkers [21,22], suggest that the dialysis procedure may trigger NETosis, thereby contributing to develop systemic microinflammation. Given the well-established association between dialysis and accelerated atherosclerosis, it is plausible that NETosis, as a shared pathological process, significantly contributes to the progression of atherosclerosis in dialysis patients [23,24].

### 2.3. The Clinical Significance of Biocompatibilities

Based on the above considerations, we propose that contemporary issues in the biocompatibility of hemodialysis lie in its potential to amplify subclinical inflammation associated with underlying disorders in individual dialysis patients (Figure 1). Activation of the complement system during dialysis is not fully suppressed, even with the use of synthetic polymer-based dialyzers. Complement component C5a, generated via the alternative pathway, mediates neutrophil activation and enhanced chemotaxis; together with neutrophil clustering and NETosis, these processes are hypothesized to exacerbate pre-existing inflammatory lesions.

For instance, in the well-recognized pathophysiological condition known as inflammation–atherosclerosis–malnutrition (MIA) syndrome, observed in dialysis patients [25], both endogenous (such as comorbidities) and exogenous factors (such as dialysis-related biocompatibility) contribute to the inflammatory milieu. Among these, the latter is thought to act as an amplifying factor of the former. Other clinical conditions involving both types of inflammatory factors include dialysis-related amyloidosis, pruritus, and fatigue, major patient-reported outcomes. Dialysis-related pruritus and fatigue, which are closely linked to patient prognosis [26,27], highlight the clinical importance of biocompatibility in dialysis therapy.

## 3. Core Mechanisms of Biocompatibility: Complement Activation, Neutrophil Stimulation, and the Interleukin-8 Pathway

### 3.1. Fundamental Biological Responses in Biocompatibility: The Roles of Complements and Neutrophil Activations

Since dialysis materials are inherently foreign to the human body, it is inevitable that the host will exhibit immune responses upon contact with these materials (Table 1). Among the various biological responses observed, complement activation and neutrophil stimulation are presumed to be the most fundamental and upstream mechanisms based on the principles of innate immunity [28]. These components constitute the body’s primordial defense systems against foreign bodies, damaged cells, and viruses, functioning ubiquitously throughout the host [28]. Accordingly, their activation plays a central role in the biological responses associated with biocompatibility.

In particular, we propose that NETosis, a specialized neutrophil response, is critically involved in this context. In the following section, we will provide an overview of the molecular biological mechanisms underlying biocompatibility, focusing on the responses of complement and neutrophils. Moreover, we also discuss interleukin-8 (IL-8), a key chemokine that promotes neutrophil chemotaxis and, according to recent studies, plays an important role in inducing NETosis. This overview incorporates our working hypotheses regarding these pathways.

### 3.2. Unresolved Issues: Complement Activation by Hemodialysis Membranes

The complement system is one of the most primitive components of the innate immune system and comprises a group of serum proteins. It may play crucial roles in host defense by directly attacking pathogens and enhancing their clearance through phagocytosis by immune cells such as monocytes, macrophages, and neutrophils [29]. Complement activations may also occur via three distinct pathways: the classical pathway (triggered by antigen–antibody complexes), the lectin pathway (initiated by recognition of carbohydrate structures on microbial surfaces), and the alternative pathway (spontaneous activation upon contact with pathogen surfaces). In hemodialysis, complement activation is primarily triggered via contact with foreign surfaces, with the alternative pathway being the dominant route of activation [29]. This leads to the generation of anaphylatoxins such as C3a and C5a, which in turn induce neutrophil activation, cytokine release, and increased vascular permeability. The degrees of complement activations vary depending on the material of the dialyzer membrane [30]. Cellulose-based membranes, such as regenerated cellulose and Cuprophan, which are some of the earliest-used dialysis membranes, are known to strongly activate complements. This is thought to be due to the high reactivity of their hydroxyl groups (^–^OH), which, due to their low hydrophobicity, promote strong nonspecific adsorption of complement proteins and facilitate the activation of C3 and C5. In contrast, current dialysis practice primarily employs synthetic polymer membranes, which are expected to reduce excessive complement activation. However, such activation is not completely suppressed [31,32]. Thus, even with advanced materials, complement activations remain an important and unresolved issue in current dialysis therapy.

### 3.3. Neutrophil Responses During Hemodialysis

**Figure 2:**
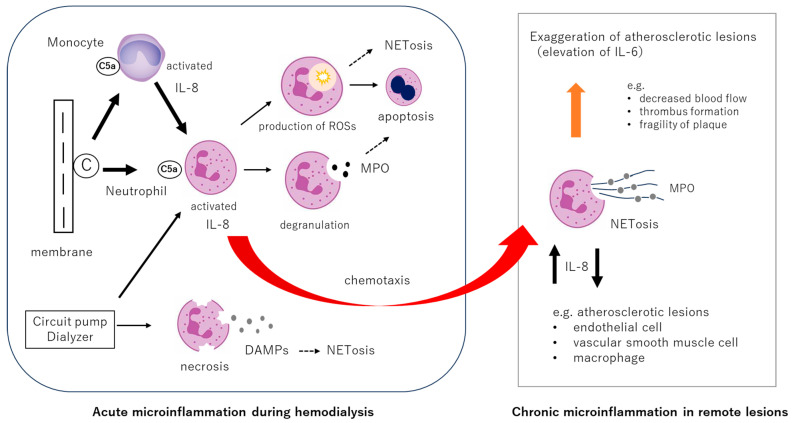
Hemodialysis biocompatibility and microinflammation—molecular biological mechanisms. The dialysis membrane activates the complement system via the alternative pathway, leading to the production of C5a, which in turn activates neutrophils and monocytes. Activated neutrophils enhance local oxidative stress through the production of reactive oxygen species (ROS) via respiratory burst and degranulation (including the release of myeloperoxidase [MPO]). Neutrophils with enhanced chemotactic activity accumulate at sites such as atherosclerotic lesions where IL-8 is expressed, and amplify local inflammation and IL-6 production via NETosis. During dialysis, IL-8 production in neutrophils is stimulated, promoting the further clustering of neutrophils at lesion sites. In addition, dialysis materials can activate neutrophils independently of the complement system. Furthermore, damage-associated molecular patterns (DAMPs) released from neutrophils due to mechanical stress can also trigger NETosis.

Two primary early responses of neutrophils are presumed to occur during hemodialysis: activation via complement component C5a and mechanical fragmentation (Figure 2). Following C5a-mediated activation, neutrophils may undergo various downstream reactions: (1) enhanced activity of NADPH oxidase leads to a respiratory burst, resulting in the increased production of reactive oxygen species (ROS) such as superoxide anions and hydrogen peroxide [30]; (2) degranulation occurs, releasing enzymes including elastase and myeloperoxidase (MPO) [33,34]; and (3) neutrophil chemotaxis is augmented, facilitating migration to distant inflammatory sites. On the other hand, complement-independent neutrophil responses may include (4) mechanical disruption due to turbulent flow and pressure from dialysis circuit pumps, resulting in the release of DAMPs from fragmented neutrophils [35]. It is well known that peripheral neutrophil counts transiently decrease during dialysis [36]. This has traditionally been attributed to redistribution, such as accumulation within alveolar capillaries. Our previous observations suggest an increase in neutrophil apoptosis during dialysis and a reduction in neutrophil counts post-treatment [37]. This may reflect the terminal fate of neutrophils that have undergone sterile respiratory bursts in response to dialysis stimuli, implying that the absolute number of functionally competent neutrophils is diminished during the procedure. Whether the depletion of healthy neutrophils due to hemodialysis impairs the host’s fundamental immune defenses remains unclear, and this issue warrants further investigation.

### 3.4. Neutrophil NETosis and Interleukin-8 (IL-8)

A representative chemokine, interleukin-8 (IL-8), is primarily produced by monocytes, macrophages, endothelial cells, and neutrophils. The cytokine may be rapidly synthesized within a few hours and plays a critical role in the early phase of inflammation by promptly activating and recruiting neutrophils to sites of injury or infection, thereby enhancing local immune responses [38].

**Figure 3:**
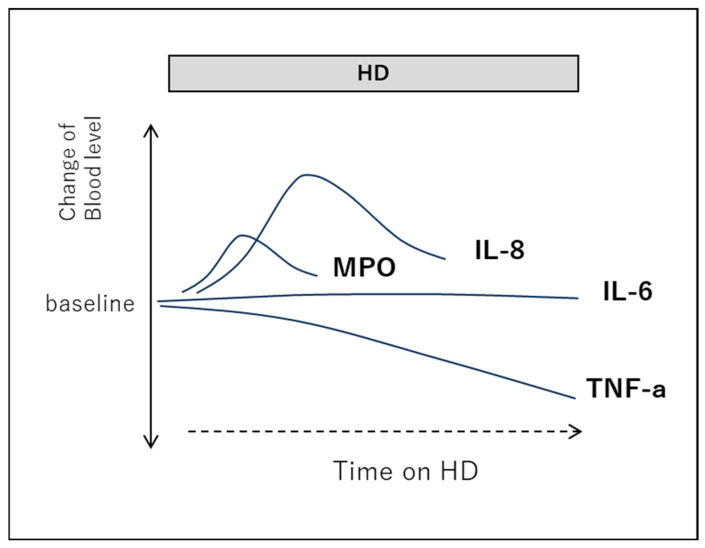
Temporal changes in cytokines and chemokines during hemodialysis (Image). IL-6: interleukin-8, IL-8: inteleukin-8, MPO: myeloperoxidase, TNF-α: tumor necrosis factor-α [39,40,41,42,43].

In the context of hemodialysis, it has been reported that while the levels of IL-1 and IL-6 remain relatively unchanged and tumor necrosis factor-alpha (TNF-α) tends to decrease, IL-8 and MPO levels increase during the dialysis session (Figure 3) [39,40,41,42,43]. These elevations may be likely initiated by complement component C5a, which activates neutrophils and monocytes upon contact with hemodialysis membranes.

It is known that non-biological materials derived from petrochemical-based synthetic polymers—such as polyethylene, polyurethane, and polystyrene—can also induce the production of IL-8 [44]. Although these materials do not contain pathogen-associated molecular patterns (PAMPs), they may activate innate immune cells indirectly through DAMPs released from stressed or damaged host cells, or through the adsorption and structural modification of self-proteins on material surfaces [45,46,47]. These stimuli engage pattern recognition receptors (PRRs), such as Toll-like receptors (TLRs) and NOD-like receptors (NLRs), leading to the secretion of proinflammatory cytokines, including IL-8, which promotes sterile inflammation [48]. Hemodialysis membranes and tubing composed of synthetic polymers have been shown to activate immune cells and stimulate the production of inflammatory mediators, including IL-8 [49]. Thus, IL-8 is not only a marker of microbial infection but also an important biomarker of host responses to non-biological materials, making it highly relevant in the evaluation of biomaterial biocompatibility [43,50].

The in vivo behavior of activated neutrophils remains incompletely understood. Given that IL-8 is also produced by neutrophils undergoing NETosis, it is possible that these activated neutrophils may contribute to the recruitment and clustering of additional neutrophils. NETs comprise various proteolytic enzymes, such as, MPO, neutrophil elastase and cathepsin G. These components exert direct cytotoxic effects on surrounding tissues and cells, while simultaneously amplifying inflammatory responses through the induction of proinflammatory cytokines such as IL-6. Moreover, tissue factor and activated elastase embedded within NETs serve as potent activators of the coagulation cascade. In parallel, NETs directly activate coagulation factor XII (Hageman factor), thereby enhancing the intrinsic coagulation pathway and promoting thrombus formation [51]. Additionally, NET-derived histones exhibit marked cytotoxicity, inducing endothelial cell apoptosis and necrosis [52]. Reactive oxygen species generated during NET formation further impair endothelial function by suppressing nitric oxide (NO) synthesis, resulting in reduced vasodilatory capacity. Collectively, NET-mediated endothelial injury leads to increased vascular permeability, heightened thrombogenicity, and accelerated progression of atherosclerosis.

Studies on the atherosclerosis have demonstrated the accumulation of NETosing neutrophils at inflammatory and atherogenic sites [53]. Moreover, NETotic neutrophils are suggested to stimulate IL-8 production through the TLR9 on macrophages, further promoting neutrophil clustering [54]. These findings suggest that neutrophil activations induced by hemodialysis may exacerbate distant inflammatory or atherosclerotic lesions by promoting the accumulation of activated neutrophils at these sites. Indeed, this mechanism may underlie the observed reduction in cerebral blood flow during dialysis in elderly patients with cerebrovascular atherosclerosis [55], highlighting the need for further investigation into the role of IL-8 and NETosis in remote organ dysfunction associated with hemodialysis.

## 4. Impact of MPO, NETosis, and IL-8 on Patient Prognosis

So far, comprehensive data regarding the clinical impact of neutrophil activation, NETosis, and IL-8 remain limited, and current evidence from existing studies is summarized below (Table 2a–c).

### 4.1. Myeloperoxidase (MPO)

MPO is a potent oxidative stress-inducing enzyme released by neutrophils, catalyzing the production of hypochlorous acid (HOCl) from hydrogen peroxide [65]. Excessive MPO activity contributes to endothelial injury and the progression of atherosclerosis. Numerous studies in non-dialysis populations have reported associations between plasma MPO levels, all-cause mortality, and cardiovascular risk. One recent study found that every 100 pmol/L reduction in plasma MPO levels was associated with a 5% decrease in five-year mortality [66]. In hemodialysis patients [56,57,58,59], elevated plasma MPO levels have been shown to correlate with other inflammatory markers [57], and even after adjustment for confounding variables, high MPO levels remain an independent risk factor for all-cause mortality [56,58,59] and cardiovascular mortality [58].

### 4.2. NETosis

Biomarkers such as plasma MPO–DNA complexes, nucleosomes, and cell-free DNA (cfDNA) have been used to evaluate NETosis. Several studies have shown increased levels of cfDNA before and after dialysis sessions [17,21]. Elevated levels of nucleosomes and neutrophil elastase were associated with impaired vasodilation [61], and soluble intercellular adhesion molecule-1 (sICAM-1, CD54) has been shown to correlate positively with NETs [21]. Nucleosome levels also correlate with MPO levels [61]. Moreover, post-hemodialysis cfDNA levels have been identified as independent predictors of mortality [60,61,62,67,68].

### 4.3. Interleukin-8 (IL-8)

Studies evaluating the prognostic significances of IL-8 in dialysis patients are limited. Small-scale investigations have reported correlations between IL-8 and inflammatory markers such as C-reactive protein (CRP) and IL-6, and IL-8 has been identified as an independent predictor of all-cause mortality and cardiovascular events [63]. Notably, a recent study evaluating 92 candidate proteins reported that only three proteins, including IL-8, were associated with all-cause mortality, cardiovascular death, and cardiovascular event incidence [64].

Together, these findings suggest that neutrophil activation triggered by complement during dialysis plays a central role in the biological response related to biocompatibility. Neutrophil responses observed during dialysis include respiratory burst, ROS production, degranulation, mechanical fragmentation DAMP release, and NETosis. However, the intensity, kinetics, and interrelationships among these responses remain largely unknown. From the perspective of microinflammation, NETosis may be a critical contributor to biocompatibility-related pathophysiology. Further investigation is warranted. Moreover, neutrophil responses can be triggered even under endotoxin-free, sterile conditions, suggesting a potential for unnecessary neutrophil consumption. The immunological consequences of such repetitive and quantitatively excessive neutrophil activation remain unclear, and this issue should be further explored, particularly in the context of infection susceptibility and immune defense in dialysis patients.

## 5. Therapeutic Strategies: Current Approaches and Future Possibilities

**Figure 4:**
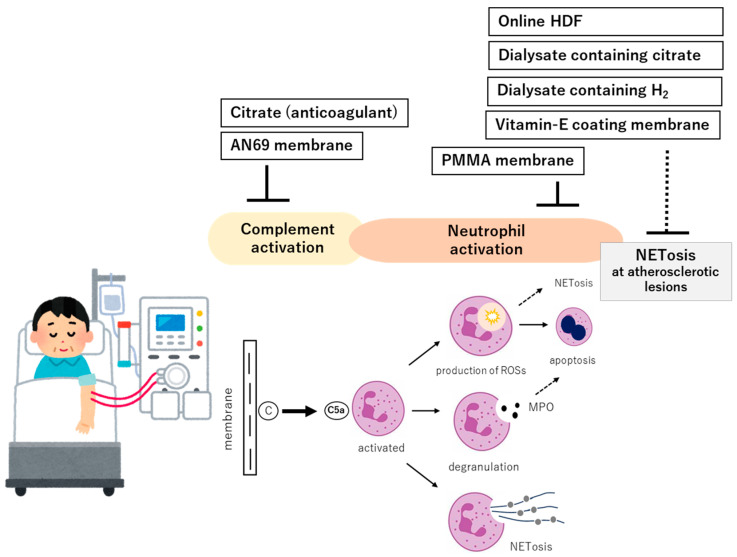
Strategies for improving the biocompatibility of hemodialysis (including potential approaches). As fundamental strategies to enhance the biocompatibility of dialysis, two principal approaches can be identified: (1) inhibition of complement activation and (2) suppression of downstream proinflammatory mediators generated by neutrophil activation. For the former, complement adsorption or inhibition strategies are considered, such as the use of AN69 membranes or citrate-based anticoagulation, both of which have demonstrated efficacy in attenuating complement cascade activation. For the latter, targeted interventions include the attenuation of oxidative stress induced by neutrophil-derived myeloperoxidase (MPO) and reactive oxygen species (ROS) through agents such as citrate or molecular hydrogen (H_2_), and the removal or adsorption of proinflammatory cytokines produced by neutrophil-monocyte lineage cells, as achieved with modalities like on-line hemodiafiltration (HDF) or polymethylmethacrylate (PMMA) membranes. Collectively, these measures are expected to mitigate neutrophil extracellular trap (NET) formation at distant inflammatory foci, thereby reducing chronic microinflammation and its systemic consequences.

In the context of complement activation, neutrophil stimulation, and the IL-8 pathway, therapeutic strategies have primarily focused on the inhibition of complement activation, suppression of neutrophil activation, and reduction in inflammation and oxidative stress, as well as downregulation of IL-8 production. The following section summarizes current therapeutic approaches and their future potential (Figure 4).

### 5.1. Dialyzers with Complement Adsorptive or Inhibitory Properties

The extent of complement activation varies depending on the material used for dialysis membranes. Cellulose-based membranes such as regenerated cellulose and Cuprophan, which are among the earliest-materials used, are known to strongly activate complements and are now rarely employed. In response to this issue, modified cellulose membranes were developed to suppress complement activation by substituting the hydroxyl groups on cellulose with more hydrophilic or chemically stable moieties [69,70]. Currently, synthetic polymer membranes are widely used in clinical practice, such as polysulfone (PS), polyethersulfone (PES), polymethyl methacrylate (PMMA), and polyacrylonitrile (AN69), which are supposed to be more biocompatible. Among them, PMMA presents a high rate of adsorption of cytokines (i.e., IL-6), and can restore adaptive immune balance by enhancing clearance of soluble CD40, a natural antagonist of the CD40/CD40 Ligand signaling that acts by inhibiting immunoglobulin production by B cells [71]. AN69 membrane, a layered-type dialyzer, has been reported to possess high complement-adsorptive capacity, reducing the production of complement-derived anaphylatoxins such as C3a and C5a [72,73].

Another material, vitamin E-coated (CLEE) membranes, have demonstrated significantly lower C3a generation compared to conventional cellulose (CLSS) membranes, along with reduced expression of the neutrophil activation marker CD11b and decreased release of MPO [74]. Moreover, CLEE membranes have been shown to suppress superoxide anion production by neutrophils, decrease the generation of hydroxyl radicals, which is a marker of oxidative stress [75], and downregulate the expression of oxidative stress-related proteins such as p22phox, PAI-1, and phosphorylated ERK1/2, while upregulating the antioxidant enzyme HO-1 [76]. These findings collectively suggest that vitamin E membranes may suppress neutrophil activation via complement inhibition. Furthermore, CLEE membranes have been associated with decreased IL-6 levels during dialysis [77], implying attenuated activation of neutrophils, lymphocytes, and monocytes. In comparisons between vitamine E-coated PS (PS-VitE) and standard PS membranes, while both exhibited comparable effects on complement activation and cytokine levels (including IL-1β and IL-8), the PS-ViE membrane resulted in reduced neutrophil elastase release and significantly lower levels of oxidative stress markers such as methemoglobin, lipid peroxides, and ROS [78]. These finding suggested that although vitamin E-coated dialyzers may not demonstrate a direct suppressive effect on complement activation, they appear to reduce oxidative stress triggered by activated neutrophils, thereby potentially mitigating secondary systemic microinflammation. However, no clinical studies to date have assessed these effects with hard clinical endpoints, and the broader clinical impact remains to be elucidated [79].

### 5.2. Dialysis Modality

#### 5.2.1. Online Hemodiafiltration

Online hemodiafiltration (OL-HDF) may utilize a large volume of substitution fluid to effectively remove medium- to high-molecular-weight uremic toxins, such as β2-microglobulin and cytokines. As a result, improvements in various inflammatory markers (IL-6, IL-8, TNF-α, and hsCRP) and oxidative stress markers (MDA, 8-OHdG, and d-ROM) have been reported [80,81]. Changes in the expression of atherosclerosis-related factors, such as reductions in VEGF-C and PDGF-AA and an increase in apolipoprotein E, have also been observed [82]. These mechanisms are suggested to be involved in a reduction in proinflammatory monocytes (CD14^+^CD16^+^) [83]. Reports with regard to complement activation, MPO, and NETosis remain limited [84,85,86]. As for complement activation, the type of membrane used may influence the outcome, and the direct effect of OL-HDF itself remains inconclusive. While sufficient data are lacking on the effect of OL-HDF on neutrophil MPO release, existing evidence suggests that OL-HDF does not suppress MPO elevation [84]. In contrast, some studies have reported that OL-HDF attenuates NETosis [85,86]. Conclusively, OL-HDF, with its high solute clearance capacity, appears to facilitate the removal of certain amounts of cytokines and chemokines, thereby alleviating subclinical inflammation. However, it remains unclear whether OL-HDF is superior to conventional HD in terms of modulating complement activity and neutrophil activation. Although several studies have demonstrated the prognostic benefits of OL-HDF, and its clinical utility is considered well-established [87,88,89], the extent to which improvements in biocompatibility contribute to these outcomes remains uncertain.

#### 5.2.2. Extended-Hour Hemodialysis

In extended-hour hemodialysis, prolonged contact time between blood and the hemodialysis membrane necessitates the use of highly biocompatible membranes and dialysis systems. Meta-analysis for mortality revealed no significant improvement by extended-hour hemodialysis [90]. Although the effects of extended-hour hemodialysis on patient survival and its association with biocompatibility have not been thoroughly investigated, one possible area of interest is whether low-flow dialysis, employed in extended-hour dialysis, suppresses neutrophil fragmentation and influences of DAMPs. These potential effects warrant further investigation. Additional studies are needed to clarify these mechanisms and their clinical implications.

#### 5.2.3. Cool Dialysate Hemodialysis

Cool dialysate hemodialysis is a therapeutic approach aimed at preventing intradialytic hypotension and improving cardiovascular stability [91]. Hypothermic conditions have been shown to suppress the activation of neutrophils and monocytes, suggesting that dialysate temperature may influence biocompatibility. One study reported that reducing the dialysate temperature to 20 °C attenuated the decline in leukocyte counts during dialysis [92]. In a preliminary investigation, plasma concentrations of IL-1, IL-2, IL-8, IL-12, and TNF-α were measured during dialysis sessions conducted with dialysate temperatures of 37 °C and 35 °C; however, no statistically significant differences were observed between the two temperature settings [93].

#### 5.2.4. Electrolyzed Water Hemodialysis

Electrolyzed water hemodialysis refers to a modality of hemodialysis utilizing dialysate containing molecular hydrogen (H_2_), as previously investigated by the authors’ research group [94]. This treatment has been associated with reductions in plasma monocyte chemoattractant protein-1 (MCP-1), a decrease in the oxidized albumin ratio (an oxidative stress marker), and lowered levels of oxidized proteins such as malondialdehyde (MDA) [95]. Interestingly, MPO levels were found to decrease in patients with initially high baseline values, while an increase was observed in those with low baseline values, suggesting a dual response. Given that H_2_-containing dialysate has been shown to inhibit neutrophil injury and potentially suppress neutrophil NETosis, it may attenuate intradialytic neutrophil fragmentation and the excessive activation of neutrophils. In clinical studies, this modality significantly reduced composite endpoints, namely all-cause mortality, cardiovascular disease (CVD) events, and lower limb amputations, compared to conventional HD [96]. Although the relationship between this therapy and biocompatibility remains unclear, it is postulated that H_2_ may exert its effects by directly scavenging hydroxyl radicals [97] and through activation of the Nrf2 pathway, leading to antioxidative and anti-inflammatory effects [95]. These mechanisms suggest that electrolyzed water hemodialysis may exert broad anti-inflammatory effects downstream of neutrophil activation.

#### 5.2.5. Expanded HD and Daily HD

Expanded HD was developed with the goal of enhancing the removal of medium- to large-molecular-weight uremic toxins, and compared to conventional HD, it enables more efficient clearance of a broader range of solutes. By employing medium cut-off (MCO) membranes, the removal of substances with molecular weights of approximately 500 to 60,000 Da—such as β2-microglobulin, inflammatory cytokines, and advanced glycation end products (AGEs) like pentosidine—is significantly increased [98]. Compared to OL-HDF, expanded HD requires less substitution fluid, making it potentially more widely applicable. There is also a suggestion that it may help prevent cardiovascular events by mitigating chronic inflammation [99]. However, its effects on biocompatibility remain unclear, and further investigation is warranted.

In Daily HD, frequent dialysis sessions are expected to improve uremic toxin control. In fact, clinical studies have reported benefits such as the prevention of heart failure [100]. However, from a biocompatibility perspective, the body is exposed to repeated stimulation, and there is currently insufficient data on this aspect. Further research is necessary.

### 5.3. Citrate as Anticoagulant

Several studies have reported on the relationship between heparin and MPO. Both unfractionated heparin (UFH) and low-molecular-weight heparin (LMWH) have been shown to promote the release of MPO from neutrophils [101]. This effect is thought to result from heparin-induced neutrophil activation, which stimulates MPO secretion. Additionally, heparin has been reported to mobilize MPO bound to the vascular endothelium into the circulating blood [102]. As a consequence, plasma MPO levels may increase, potentially enhancing oxidative stress and inflammatory responses.

Citrate exerts its effect by chelating calcium and magnesium ions, thereby inhibiting activation of the complement cascade and suppressing the formation of C3a, C5a, and the membrane attack complex [103]. It also has the potential to inhibit activation of neutrophils and platelets, consequently reducing the release of proinflammatory cytokines [104]. Furthermore, citrate has been shown to suppress the release of MPO [84]. Through these mechanisms, the application of citrate may improve biocompatibility during hemodialysis. Dialysate formulations in which acetate is replaced with citrate have been developed, and clinical trials have suggested that citrate-containing dialysate may enhance biocompatibility compared to acetate-containing solutions by attenuating inflammatory markers and NETosis [105,106].

## 6. Future Perspectives on Improving Biocompatibility

Improving biocompatibility involves mitigating the body’s natural immune response to foreign substances. However, excessive suppression of such biological responses may impair the host’s physiological immune defense mechanisms. Indeed, it has been reported that neutrophil function, including NETosis in response to bacterial stimuli, is reduced in dialysis patients compared to healthy individuals [107]. Moreover, the use of citrate in continuous renal replacement therapy has been associated with an increased risk of infections [108]. In this context, it is crucial to identify clinical scenarios where enhanced biocompatibility may confer the greatest medical benefit. This is particularly relevant in patients with comorbid atherosclerotic disease, including those with coronary artery disease and peripheral arterial disease. By integrating current evidence and applying it within the framework of personalized medicine, it may become possible to deliver optimal hemodialysis therapy tailored to each patient, emphasizing high biocompatibility. However, such approaches must also be cost-effective from a health economics perspective. The following sections outline future opportunities and challenges in improving biocompatibility, focusing on pharmacological interventions, dialysis techniques, and dialyzer innovations.

### 6.1. Potential for Improvement Through Pharmacological Agents: Citrate-Based Anticoagulation

Citrate anticoagulation is considered safer in patients at high risk of bleeding. In addition, it suppresses complement activation and leukocyte activation, thereby attenuating inflammatory responses. Wider adoption is anticipated in the future.

### 6.2. Development and Dissemination of Biocompatible Membranes and Bioactive Dialysates

#### 6.2.1. Development of Highly Biocompatible Membranes

Vitamin E-coated membranes and AN69 membranes have been shown to effectively reduce oxidative stress and inflammatory cytokines. Notably, vitamin E-coated membranes have demonstrated suppression of MPO and IL-8 production. However, challenges remain: AN69 membranes are unsuitable for OL-HDF due to their limited permeability, and vitamin E-coated membranes are relatively expensive. Overcoming these limitations is essential for broader clinical use.

#### 6.2.2. Citrate-Containing and H_2_-Enriched Dialysates

Dialysates containing citrate or H_2_ are expected to exert anti-inflammatory and antioxidative effects. Combining these dialysates with OL-HDF could enhance their efficacy. Although clinical evaluations of hydrogen-enriched dialysates have so far been limited to Japan, over 3000 patients were estimated to be receiving this therapy as of 2025. Given that H_2_ is a safe antioxidant, further clinical development may expand its use, and it holds promise as a dialysis treatment applicable across a wide patient population.

### 6.3. Utilization of Technology

Integrated management of biometric data using artificial intelligence (AI) could open up new possibilities. The real-time monitoring of biomarkers such as MPO, IL-6, and CRP may enable us to personalized optimization of dialysis conditions. If technologies are developed to allow AI to automatically adjust anticoagulant dosage such as citrate administration and ultrafiltration rates, it would become feasible to achieve dialysis treatments with significantly enhanced biocompatibility.

## 7. Conclusions

Contemporary issues related to the biocompatibility of hemodialysis lie in its potential to amplify subclinical inflammation associated with underlying disorders in individual dialysis patients, with NETosis potentially playing a pivotal role in its primary pathophysiology. In this context, enhancing biocompatibility in hemodialysis is essential for maintaining the long-term health of dialysis patients. Given the essential innate immune responses triggered during hemodialysis, complement activation, NETosis, and IL-8 are considered key surrogate markers for evaluating improvements in biocompatibility. Advancements across multiple domains—including pharmacological agents, membrane materials, and dialysis modalities—should target these surrogates. Among these, considering applicability to real-world clinical practice, it is particularly important to investigate the impact of convection volume on the temporal changes in surrogate markers in high-volume OL-HDF, and expanded HD, as well as the effects of citrate- and H_2_ -enriched dialysis solutions. Optimizing biocompatibility is crucial for mitigating cardiovascular morbidity and mortality in dialysis patients, and approaches aimed at suppressing the innate immune response during hemodialysis are expected to contribute to the development of novel standards of care in extracorporeal blood purification.

## Figures and Tables

**Table 1:** Hemodialysis and Biological Responses.

Source	Products	Influence/Symptoms
Ethylene oxide gas sterilization		First use syndrome, asthma
DEHP, a phthalate plasticizer	endocrine disruptor	endocrine disruption
Complements	C3a, C5a, Membrane Attack Complex	activation of neutrophil and monocyte
Neutrophil	ROSs (hydroge peroxide, superoxide anion)	endothelial cell damage, oxidative stress
	degradation (myeloperoxidase, elastase, et al.)	
Platelet	thromboxane A_2_, Platelet-Derived Growth Factor, prostaglandins	platelet aggregation, thrombosis, atherosclerosis
Basophil·Mast cell·Eosinophil	histamine, leukotriene	hypotension, bronchospasm
Monocyte	Interleukin (IL)-1, IL-6, IL-8,	dialysis-related amyloidosis, microinflammation
	Tumor Necrosis Factor-α, Interferon	
Coagulation–Fibrinolysis system	Factor XIIa	blood chamber coagulation
Kinin–Kallikrein System	bradykinin	anaphylactoid reaction

**Table 2:** Mortality risk of myeloperoxidase (a), NETosis (b), and Interleukin-8 (c) in patients on hemodialysis.

Author (Year) Reference	Number of Patients (Observation Periods)	Results
**(a) Myeloperoxidase (MPO)**		
Kalantar-Zadeh (2006) [56]	356 (retrospective analysis)	Hazard Ratios (HRs) for death
		1.14 (1.03–1.26: 95%CI); P 0.01 each 1000-pmol/L of plasma MPO, 1.82 (1.07–3.10: 95%CI) in the highest as compared with the middle tertile
Wang (2010) [57]	236 (3 years)	A doubling in plasma MPO level: increases of 46% (1.02–2.08; 95%CI) in mortality, and 60% (1.17–2.18) in cardiovascular events.
Zuo (2022) [58]	347 (60 months)	HRs for death: 1.000035 (1.000020–1.000051: 95%CI) by univariate, and 1.000033 (1.000018–1.000049: 95%CI) by multivariate analysis
Liberale (2024) [59]	1182 (median 2.9years)	HRs for all cause for mortality: 1.26 (1.11–1.42: 95%CI), and for cardiovascular death: 1.19 (1.01–1.41: 95%CI)
**(b) NETosis**		
Tovbin (2012) [60]	31 (42 months)	HR for all-cause of death of cell free DNA higher than 850 ng/mL: 8.0 (2.3–28.5: 95%CI)
Kim (2020) [61]	281	the nucleosome Q4 group had significantly increased all-cause and cardiovascular mortality compared to the Q1–3 groups
Einbinder (2020) [62]	153 (46 months)	HR for mortality of post-HD cfDNA: 1.92 (1.03–3.56: 95%CI),
		OR for mortality of post-HD cfDNA: 4.61(1.45–14.66: 95%CI) by 1 year, 4.36 (1.63–11.66) by 2 years, and 6.22 (2.2–17.59) by 3 years.
**(c) Interleukin-8**		
Panichi (2006) [63]	76 (18 months)	the strongest independent predictor of all-cause and cardiovascular by stepwise regression analysis
Wu (2022) [64]	331 (5 years)	HRs for all-cause mortality: 1.29 (1.11–1.59: 95%CI), for cardiovascular disease-mortality: 1.34 (1.02–1.76), and all vascular events: 1.33 (1.11–1.59)
